# The role of the axillary Impella 5.0 device on patients with acute cardiogenic shock

**DOI:** 10.1186/s13019-020-01251-7

**Published:** 2020-08-14

**Authors:** Saeed Tarabichi, Hirohisa Ikegami, Mark J. Russo, Leonard Y. Lee, Anthony Lemaire

**Affiliations:** Division of Cardiothoracic Surgery, Department of Surgery, RUTGERS-Robert Wood Johnson Medical School, 125 Paterson Street, New Brunswick, NJ 08903 USA

**Keywords:** Cardiogenic shock, Heart, Device

## Abstract

**Background:**

Acute cardiogenic shock is associated with high mortality rates. The Impella device is a microaxial left ventricular assist device that can be inserted through the axillary artery. The purpose of our study is to determine the role of the Axillary Impella devices on patients with acute cardiogenic shock.

**Methods:**

A retrospective chart review was conducted to identify patients who underwent Axillary Impella device placement for acute cardiogenic shock from January 1st, 2014 to September 30th, 2018 at a single institution. In-patient records were examined to determine duration of device, length of stay (LOS), postoperative complications, and 30-day in-hospital mortality.

**Results:**

A total of 40 patients, who were primarily men (*N* = 29) with a mean age of 61.2 ± 10.7 years old, underwent Axillary Impella placement for cardiogenic shock. The primary reasons for implant were (1) required upgraded support from an Impella CP or intra-aortic balloon pump (iabp) to Impella 5.0, (2) to treat left ventricular (LV) distention for patients on extracorporeal mechanical oxygenation (ECMO), and (3) to provide longer term support and allow for mobilization of the patients in whom a device was already indwelling. Twenty-three of the patients had previous devices already in place including a Femoral Impella CP device or an iabp and 9 patients were on ECMO support. The duration of the device was 21.05 ± 17 days with the LOS of 40.8 ± 28 days for those patients. Seventeen of the patients went on to additional surgery including (1) Heartmate 3 device placement (*N* = 6), (2) other cardiac procedures such as surgical revascularization (*N* = 9), and orthotopic heart transplantation (*N* = 2). A total of 21 patients of the 40 (52%) died during their hospitalization with 7 patients (17%) having complications related to the Impella device. These complications included right arm ischemia or neuropathy (*N* = 3) and Impella malfunction requiring device replacement (*N* = 4). The majority of these devices were placed in the right axillary artery (*N* = 38) versus the left axillary artery (*N* = 2).

**Conclusions:**

A total of 58% (*N* = 23) of the study patients had previous mechanical support and 23% (*N* = 9) were on ECMO demonstrating the severity of disease and accounting for the high mortality. The Axillary Impella device allows for a minimally invasively placed device that is durable with a mean duration of 3 weeks. The Axillary artery Impella 5.0 provides upgraded full cardiac support while allowing for mobilization of the patient. In addition, it treats LV distention in patients on ECMO while avoiding sternotomy. Finally, the Axillary Impella provides time for decision making for explant, additional therapy with either long-term devices or orthotopic heart transplant.

## Introduction

Cardiogenic shock (CS) is commonly defined as a physiologic state in which cardiac function is inadequate to perfuse the tissues. If CS is not rapidly recognized and treated, tissue hypoperfusion can quickly lead to organ dysfunction and patient death [1]. The initial management of CS is medical therapy however when this fails mechanical support is often required. Although CS is often an acute issue, patients with heart failure often have a chronic condition that may warrant mechanical support. Heart failure is a critical problem and continues to rise in incidence as the population in developed countries continues to grow older. In the United States, heart failure has been identified as a growing epidemic affecting over 5 million Americans and 23 million throughout the world [2].

The Impella left ventricular assist device (Abiomed, Danvers, MA), Impella, is increasingly being used for mechanical circulatory support for acute CS [3]. It is rapidly deployed and improves heart function in patients with acute CS but also for patients with chronic heart failure. They have proven to be safe, and effective at improving hemodynamic parameters when the heart is acutely decompensated [4]. The devices can be inserted with multiple approaches but also with different types of operators. The purpose of our study is to determine the role of the axillary Impella devices on patients with acute CS and chronic heart failure.

## Methods

This is a retrospective review of consecutive patients that had an axillary Impella placed at a single academic institution. A retrospective chart review was conducted to identify all patients who underwent axillary Impella device placement for acute CS and chronic heart failure from January 1st, 2014 to September 30th, 2018 at a single institution. In-patient records were then individually examined in a chart review to assess both patient demographic information, as well as outcome. The primary outcome variable is 30-day hospital mortality rate. Secondary variables include duration of device placement, length of stay, indications for device use, post-operative complications, and ultimate outcome. The axillary Impella devices that were implanted primarily consisted of ABIOMED Impella CP or Impella 5.0.

## Results

**Fig. 1:**
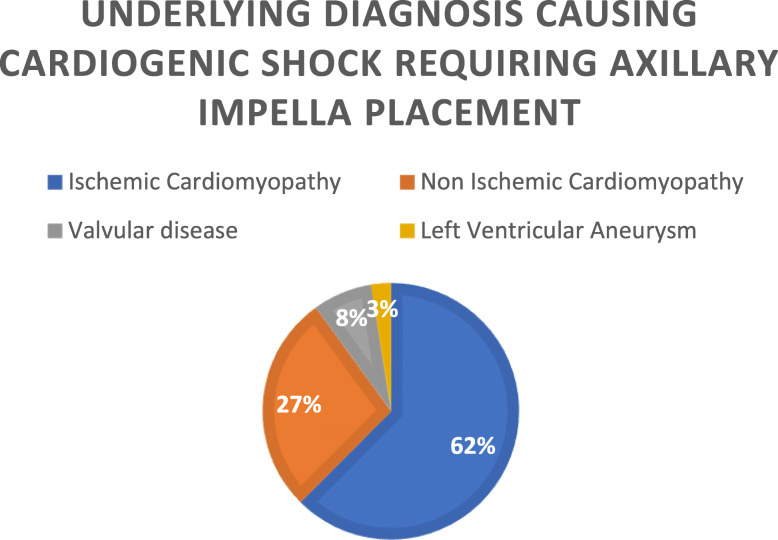
Breakdown of the underlying diagnosis requiring axillary Impella placement for this study

A total of 40 patients were identified who had the axillary Impella device inserted from January 1st, 2014 to September 30th, 2018. The patients were primarily men (*N* = 29), with a mean age of 61.2 ± 10.7 years old. The majority of these devices were placed in the right axillary artery (*N* = 38, 95%) and the remaining were placed in the left axillary artery (*N* = 2). The main indication for placement was cardiogenic shock secondary to ischemic cardiomyopathy in 62.5% of patients (*N* = 25). Other indications included non-ischemic cardiomyopathy, valvular disease, and a left ventricular aneurysm (see Fig. 1).

A large proportion of patients in the study had assist devices that were already placed prior to having the axillary Impella inserted. Twenty-three of the patients (57.5%) had a femoral Impella CP device or intra-aortic balloon pump (IABP) previously. In these patients the Impella 5.0 placed through the axillary artery served as an upgrade to the Impella CP or the IABP. A total of 9 (22%) patients were on extracorporeal membrane oxygenation (ECMO) support prior to Impella placement. The axillary Impella in these ECMO patients provided multiple benefits. First, the Impella prevents left ventricular distention, which is one of the more common problems with ECMO support. Second, the Impella 5.0 allows for a transition for these patients off full Venous-Arterial (VA) ECMO support to a device that provides 5 l of flow and avoids the well-known risk of longer term ECMO. The Impella device requires less anticoagulation than ECMO and allows for patient mobilization not possible with VA ECMO.

These findings suggest that the patients that were getting the axillary Impella were very critically ill with a mean STS score of > 8%. Furthermore, it shows that the axillary Impella is often not a device placed emergently and quickly. It requires a cutdown to the artery and anastomosis of the axillary artery to an 8- or 10- mm graft. This makes the axillary impella more distinct from the Impella CP and Impella LD. The Impella CP is more often placed by Interventional Cardiologists during percutaneous coronary intervention (PCI) or acute cardiogenic shock. Similarly, the Impella LD is placed centrally through a graft sewn to the ascending aorta as a mechanism to provide additional support or assist with coming off cardiopulmonary bypass.

The mean duration of the device was 21.05 ± 17 days with the length of stay (LOS) of 40.8 ± 28 days for those patients. Seventeen of the patients (42.5%) went on to additional surgery (See Table 1) including: (1) Longer term left ventricular assist device (LVAD) (Heartmate 2, 3, Heartware) placement (*N* = 6), (2) Surgical revascularization (*N* = 8), (3) Valve replacement (*N* = 3), and (4) orthotopic heart transplantation (*N* = 2). A total of 21 patients (52%) died during their hospitalization with 9 patients (22.5%) having complications related to the Impella device. Only 1 patient out of the 9 which suffered device related complications suffered mortality. See Table 2 for a list of Impella related complications.

**Table 1:** Outcomes of patients who underwent axillary Impella placement showing average length of stay as well as duration of Impella device usage

Outcome	Number of patients	Average hospital length of stay	Average number of days with axillary impella
**Longer term LVAD placement**	6	66.8 days (STDV = 30.1 days)	26.6 days (STDV = 19.57 days)
**Recovery after revascularization**	8	37.75 days (STDEV = 16.6 days)	16.75 days (STDEV = 6.67 days)
**Valve replacement**	3	74.6 days (STDEV = 33.5 days)	47.3 days (STDEV = 37.84 days)
**Orthotopic heart transplant**	2	43 days (STDEV = 4.24 days)	29.5 days (STDEV = 0.7 days)

**Table 2:** List showing all Impella related complications during study time period. One of nine patients who had Impella complications suffered mortality

Impella related complications (*N* = 9)	Mortality
Accidental Impella dislodgement	No
Impella malfunction requiring explant	No
Axillary exploration for hematoma	No
Impella malfunction requiring reinsert	No
Impella malfunction requiring reinsert	No
Limb ischemia requiring axillary artery decompression	Yes
Brachioplexopathy	No
Acute limb ischemia requiring embolectomy	No
Impella malfunction requiring reinsertion	No

## Discussion

Minimally invasive cardiac surgery is becoming more prevalent in the treatment of patients with heart failure or acute CS [5, 6]. Many of these patients have multiple co-morbidities that limits them from undergoing complex procedures. The axillary Impella provides an option for a left ventricular assist device that can be placed through a minimally invasive approach. The Impella provides multiple opportunities for treatment including left ventricular unloading, as compared with increased afterload in ECMO therapy and it benefits as a bridge to recovery or destination therapy. These findings have been supported by multiple studies that has compared the use of VA ECMO with Impella and VA ECMO patients alone [7, 8]. In patients with cardiogenic shock, the “ECPELLA” (ECMO and Impella) was associated with significant decreases in central venous pressure, pulmonary pressures and vasoactive medication requirements as compared to on VA ECMO [7, 8]. In addition, the use of ECPELLA was associated with greater ECMO weaning and bridging to permanent therapies [9].

There has been a growth of minimally invasive surgical procedures demonstrated by increasingly popular approaches including partial sternotomy, right or left thoracotomies, robotic and transcatheter approaches [10–12]. The Impella 5.0 provides upgraded full cardiac support (> 5 l of flow) for patients suffering from acute decompensated heart failure and cardiogenic shock. The device does not require a median sternotomy to insert and provides additional mechanical support than other devices that are placed percutaneously. The axillary Impella is an excellent option for those cardiac surgery patients, on previously placed mechanical support, who require escalation of support. A majority of patients within our dataset (58% (*N* = 23)) were upgraded from previous mechanical support. Twenty-three percent (*N* = 9) were on ECMO demonstrating the severity of disease and accounting for the high mortality. Although the Impella does not serve as a permanent solution for CS, the Impella via the minimally invasive approach allows for mechanical support that is well tolerated in even the sickest heart failure patients.

The most significant benefit to the full cardiac support provided by axillary Impella 5.0 is the ability to provide the patient with valuable extra time for decision making. It allows the medical team to plan for additional therapy with either long-term devices or orthotopic heart transplant as demonstrated by the numerous patients that went on to additional procedures in our dataset (See Table 1). In extreme cases where multi system organ failure require specialized testing or studies, an Impella device may allow for more stable transportation of a patient to hospital facilities. The Impella 5.0 device may also be placed via a superficial femoral artery cutdown, however concerns for wound complications from groin incision and the lack of mobility limits its’ use. The incidence of wound complications from groin cutdowns in vascular surgery vary but may range from 2.1 to 22.8% [13–15]. Furthermore, the incidence of access related complications to the superficial femoral artery have been reportedly as high as 11% [16] this high incidence is not seen with axillary artery cutdown. For patients who are diabetic or obese, infectious complications of a groin cutdown are a significant concern. Surgical division of lymphatic channels within the groin also pose a problem for limb lymphedema and seroma formation.

The axillary artery provides a target closer to the aortic arch in which a larger Impella device may be implanted without the significant morbidity of extremity lymphedema, groin wound infection, or seroma. We have shown that complications specifically related to the axilla were limited to 4 out of the 40 patients that were monitored. Brachioplexopathy and acute extremity limb ischemia carry a significant morbidity. Furthermore, we were able to demonstrate that the axillary Impella allows for a minimally invasive placed device that is capable of providing full cardiac support.

## Conclusion

Although, the disease burden for patients requiring increased support with axillary Impella is exceedingly high, nearly half the patients who had the Impella placed, survived their hospitalization. It is demonstrably durable with a mean duration of 3 weeks. The Impella related complication rate was 22.5%, which is not insignificant, but only 1 of those 9 patients with complications suffered a mortality. There were no wound infection complications associated with axillary artery exposure for Impella 5.0 placement. Taken together, the findings from this study suggest the axillary Impella to be the overall superior approach in patients requiring temporary advanced mechanical support.

## Data Availability

The dataset for the study are available from the Corresponding author upon request.

## Abbreviations

CS: Cardiogenic Shock
IABP: intra-aortic balloon pump
ECMO: Extracorporeal Membrane Oxygenation
VA: Venous Arterial
LVAD: Left ventricular assist device
PCI: Percutaneous Coronary Intervention
